# The changing characteristics and molecular profiles of papillary thyroid carcinoma over time: a systematic review

**DOI:** 10.18632/oncotarget.12885

**Published:** 2016-10-25

**Authors:** Huy Gia Vuong, Ahmed M.A. Altibi, Amr Hesham Abdelhamid, Phuong Uyen Duong Ngoc, Vo Duy Quan, Mohamed Yousef Tantawi, Mohamed Elfil, Tran Le Huy Vu, Ahmed Elgebaly, Naoki Oishi, Tadao Nakazawa, Kenji Hirayama, Ryohei Katoh, Nguyen Tien Huy, Tetsuo Kondo

**Affiliations:** ^1^ Department of Pathology, University of Yamanashi, Yamanashi, Japan; ^2^ Department of Pathology, Cho Ray Hospital, Ho Chi Minh City, Vietnam; ^3^ Faculty of Medicine, University of Jordan, Amman, Jordan; ^4^ Faculty of Medicine, Menoufia University, Shebin El-Kom, Menoufia, Egypt; ^5^ Pham Ngoc Thach University of Medicine, Ho Chi Minh City, Vietnam; ^6^ University of Medicine and Pharmacy, Ho Chi Minh City, Vietnam; ^7^ Faculty of Medicine, Zagazig University, Zagazig, Egypt; ^8^ Faculty of Medicine, Alexandria University, Alexandria, Egypt; ^9^ University of California, Los Angeles, CA, USA; ^10^ Faculty of Medicine, Al-Azhar University, Cairo, Egypt; ^11^ Department of Immunogenetics, Institute of Tropical Medicine, Leading Graduate School Program, and Graduate School of Biomedical Sciences, Nagasaki University, Sakamoto, Nagasaki, Japan; ^12^ Department of Clinical Product Development, Institute of Tropical Medicine, Nagasaki University, Nagasaki, Japan

**Keywords:** BRAF, RAS, RET/PTC, papillary, thyroid carcinoma

## Abstract

**Background:**

The genetic alterations of papillary thyroid carcinoma (PTC) have been reported to change over the past few decades. We performed this systematic review to further examine the trends and modifications of patient demographic, clinicopathological features and molecular profiles of PTC over time.

**Methods:**

A literature search was performed within six electronic databases to identify relevant articles. The inclusion criteria were published studies investigating *BRAF* mutations, *RET/PTC* rearrangements or *RAS* mutations in PTCs or classical PTCs. Two teams of reviewers independently screened titles and abstracts of all articles. Full texts of potential articles were read and extracted data were listed and stratified into an excel file according to country, city, institution, and surgical time period. Student t test and Pearson Chi-square were used to analyze the trends of demographic and clinicopathological features of PTC patients and the prevalence of each genetic alteration in individual institutions.

**Results:**

From 3139 articles, we included 16 articles for final analysis. Our results showed an increasing trend of *BRAF* and a decreasing trend of *RET/PTC* prevalence over time in PTCs and classical PTCs, accompanied by an older age of PTC patients, an increase in proportion of PTMC and less aggressive behaviours of tumours.

**Conclusions:**

The demographic and clinicopathological characteristics and molecular profile of PTCs have been changing over the past few decades. These modifications suggest changes in etiologies and risk factors of thyroid cancer that influence the tumorigenesis of PTCs.

## INTRODUCTION

Thyroid cancer is the most common endocrine malignancy and its rate has been steadily increasing all over the world [1, 2]. The incidence of thyroid cancer more than doubled over the past 30 years in the United States (US) [3]. Davies et al. reported that the incidence of thyroid cancer was relatively stable until the early 1990s, after which its incidence increased remarkably due to an increase in PTCs [4]. The underlying causes of this increase are controversial. One proposal for the increasing incidence of thyroid cancer says it may be the result of two coexisting processes: increased diagnostic scrutiny and an increased number of cases due to unrecognized thyroid specific carcinogens [5]. Morris et al., however, stated that improved detection does not fully explain the rising incidence of thyroid cancer, and instead, a true increase in the incidence of thyroid cancer might be due to unknown hormonal, dietary, genetic or environmental risk factors [6].

In PTCs, the most common mutations are *BRAF* mutations, which were reported in 40-90% of cases from various geographical areas [7–9]. The second most common genetic alteration in PTC are *RET/PTC* rearrangements [10]. Both single- and multi-institutional studies in Italy, Korea and the US have shown a significant and steady increase in the prevalence of *BRAF* mutations [11–13]. On the other hand, *RAS* mutations have increased sharply, and *RET/PTC* rearrangements have steadily decreased over the years [11, 13, 14]. Marthur et al. also linked the increased *BRAF* mutation prevalence with increased thyroid cancer incidence [13]. However, it is still continuously debated whether the genetic background of PTC changed or not [14]. Therefore, we conducted this systematic literature review to investigate the worldwide trends of *BRAF* mutations, *RET/PTC* rearrangements and *RAS* mutations in PTCs.

## RESULTS

### Search results

**Figure 1:**
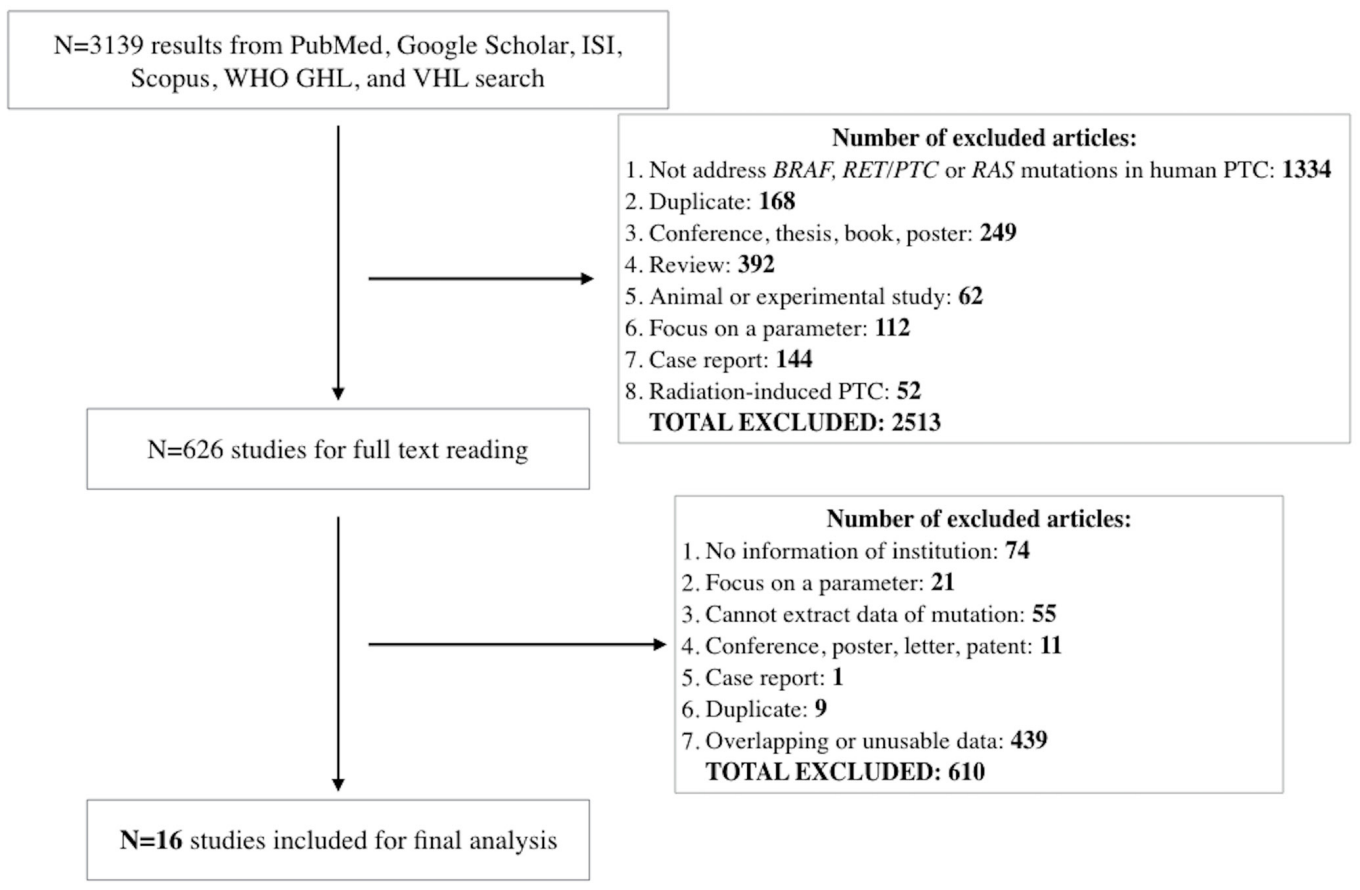
Flow diagram of study selection Abbreviations: WHO GHL, World Health Organization Global Health Library; VHL, Virtual Health Library.

We identified 3139 results from the six libraries, but only 626 articles met our selection criteria. After carefully reading full-text versions of these 626 articles, we excluded 232 more studies. Overall, we listed and stratified 394 articles containing prevalence data of at least one genetic alteration into an excel file according to country, city, institution and the time period when surgery was performed. We used this listing to facilitate further data selection for analysis as described in each section below. To avoid overlapping patient data between publications with overlapping surgical time periods of patients from the same institution, we selected studies with the larger number of patients. Our final analysis included 16 studies (Figure 1).

### Trends for *BRAF* mutations, *RET/PTC* rearrangements and *RAS* mutations in classical PTCs from individual institutions

*BRAF* mutations have been reported to be associated with variants of PTCs: high prevalence in tall cell variant and classical PTCs and low prevalence in FVPTCs. In contrast, *RAS* mutations were reported as most commonly found in FVPTCs. Therefore, we examined the trends of *BRAF* mutations, *RET/PTC* rearrangements and *RAS* mutations in classical PTCs, the most common PTC variant, to avoid bias caused by other variants. We used the following criteria to select eligible data from the stratified list: (i) the study institution had available data on *BRAF* mutations, *RET/PTC* rearrangements or *RAS* mutations in classical PTCs from at least 2 different surgical time periods, (ii) the same detection method provided by the same manufacturer was used between the surgical time periods and (iii) patients with classical PTCs from all surgical periods received the same treatment (for example, all patients underwent total thyroidectomy) or the authors did not use any criteria that could influence the characteristics of classical PTCs during patient selection in each period.

We found appropriate *BRAF* data from two US institutions (University of California at San Francisco (UCSF) [13] and University of Pittsburgh [14]), an Italian multicenter study at the Endocrine Units of Pisa/Perugia/Milan [11], a Korean medical center (Seoul St. Mary's Hospital) [15, 16], an Irish institution (St. James's Hospital) [17] and an institution from Poland (Holycross Cancer Center) [18]. Prevalence of *BRAF* mutations in classical PTCs increased significantly in the studies from all of the institutions, except Seoul St. Mary's Hospital in Korea (Table 2).

**Table 1:** Characteristics of included studies

Author / Year of publication / Country	Institution	Surgical Period	No. of cases	Mean of age	Tissue treatment method	Patient selection criteria	Detection method (Manufacturer)
Mathur/2011/US [13]	University of California at San Francisco	1991 – 1995 1996 – 2000 2000 – 2005	74 103 51	46.4 43.5 48.7	Frozen Frozen Frozen	Consecutive patients underwent initial evaluation and treatment at the institution. Thyroidectomy was performed in all cases.	BRAF, RAS: Direct sequencing (Applied Biosystem) RET/PTC: Nested PCR
Jung/2014/US [14]	University of Pittsburgh	1974 – 1985 1990 – 1992 2000 2009	62 22 24 65	37.0 ^a^ 51.0 ^a^ 50.0 ^a^ 53.0 ^a^	FFPE FFPE FFPE FFPE	Cases were chosen consecutively within each of preselected periods until the numeric target was fulfilled. No selection criteria of cPTCs were described.	BRAF, RAS: Real-time LightCycler PCR and fluorescent melting curve analysis (Roche) RET/PTC: Dual-color interphase FISH
Romei/2012/Italy [11]	Endocrine units of Pisa, Perugia and Milan	1996 – 2000 2001 – 2005 2006 – 2010	56 98 106	40.9 44.0 47.6	Frozen Frozen Frozen	Cases were neither consecutive nor selected, and the same procedures of collection were followed in the three centers. No selection criteria of cPTCs were described.	BRAF: Direct sequencing (Applied Biosystem) RET/PTC: Southern blotting
Nam/2012/Korea [18]	Seoul St. Mary's Hospital	2008 – 2009	355	46.9	FFPE	Thyroid tumor tissues were sampled from patients with PTC who underwent thyroidectomy. No selection criteria of cPTCs were described.	BRAF: Direct sequencing (Applied Biosystem)
Lee/2014/Korea [19]	Seoul St. Mary's Hospital	2011 – 2012	200	45.7	FFPE	The patients with PTC who underwent surgery at the institution during the selected time period were enrolled in this study. No selection criteria of cPTCs were described.	BRAF: Direct sequencing (Applied Biosystem)
Smyth/2005/Ireland [20]	St. James's Hospital	1982 – 2003	18	49.6	FFPE	Samples of PTC accessioned between 1982 – 2003 were analysed. No selection criteria of cPTCs were described.	BRAF: Direct sequencing (Applied Biosystem)
Kowalska/2016/Poland [21]	Holycross Cancer Center	2000-2004 2005-2009 2010-2013	152 195 254	52.0 ^a^ 53.0 ^a^ 54.0 ^a^	FFPE FFPE FFPE	Archived tumor tissue was retrieved from 785 unselected PTCs diagnosed between 2000-2013. No selection criteria of PTCs were described.	BRAF: Direct sequencing (Applied Biosystem), Allele-specific PCR and quantitative real time PCR (Qiagen)
Nakazawa/2009/Japan [22]	University of Yamanashi	2001 – 2006	14	50.4	FFPE	Surgical specimens were retrieved from the institution. A thorough review of clinical data revealed no history of radiation exposure. No selection critera of cPTCs were described.	RET/PTC: Nested PCR
Vuong/2016/Japan [23]	University of Yamanashi	2011 – 2014	59	49.3	FFPE	We selected randomly patients with primary PTC who had been initially treated at the institution. No selection criteria of cPTCs were described.	RET/PTC: Nested PCR
Lee/2013/Korea [26]	Samsung Medical Center	2008 – 2009	396	47.0	FNAB	Patients who underwent thyroidectomy for primary PTC during the time period were enrolled. No selection criteria of cPTCs were described.	BRAF: Direct sequencing (Applied Biosystem)
Kang/2013/Korea [27]	Samsung Medical Center	2010 – 2011	128	48.0	FFPE	Patients with PTC were randomly retrieved from the surgical pathology files. No selection criteria of cPTCs were described.	BRAF: Direct sequencing (Applied Biosystem)
Zheng/2013/China [24]	Tianjin Medical University Cancer Hospital	2001 – 2010	977	46.0	FFPE	A chart reviewed identified 2046 patients with PTC who underwent surgery during the time period. Among of them, 977 patients had PTMCs. No selection criteria of PTMCs were described.	BRAF: Direct sequencing (Applied Biosystem)
Lu/2013/China [25]	Tianjin Medical University Cancer Hospital	2013 – 2014	1984	45.0 ^a^	FFPE	The clinical data was reviewed on 1990 PTMCs during the time period. No selection criteria of PTMCs were described.	BRAF: Direct sequencing (Applied Biosystem)
Barzon/2008/Italy [28]	University of Padova	2005 – 2007	83	43.6	Frozen	Consecutive patients who underwent thyroidectomy at the institution in the selected period and had a pathological diagnosis of PTC. No selection criteria of PTCs were described.	BRAF: Direct sequencing (Applied Biosystem)
Pelizzo/2011/Italy [29]	University of Padova	2008 – 2009	270	48.9	FNAB	Consecutive patients within selected time period were prospectively enrolled. All patients had a sonographic single node and/or a node with suspected features who underwent US-guided FNA and subsequent thyroidectomy at the institution.	BRAF: Direct sequencing (Applied Biosystem)
Hwang/2009/Korea [30]	Samsung Medical Center	2008	135	47.0	FNAB	Consecutive patients with PTC that had been confirmed surgically were included in the study. All patients underwent ultrasound and ultrasound-guided FNA.	BRAF: Direct sequencing (Applied Biosystem) and allele-specific PCR

Abbreviations: cPTC, classical papillary thyroid carcinoma; PTMC, papillary thyroid microcarcinoma; FFPE, formalin-fixed paraffin embedded; FNAB, Fine-needle aspiration biopsy

^a^ Median value

**Table 2:** The trends of BRAF mutations prevalence of classical PTCs from individual institutions

Country	City (State)	Institution (Reference)	Prevalence of *BRAF* mutations in classical PTCs					
			Period 1	Period 2	Period 3	Period 4	*P*	
US	San Francisco (CA)	UCSF [13]	1991-1995 38/74 (51.4%) DS	1996-2000 44/103 (42.7%) DS	2001-2005 45/51 (88.2%) DS		**<0.001**	
US	Pittsburgh (PA)	Univ. of Pittsburgh [14]	1974-1985 31/62 (50.0%) FMCA	1990-1992 16/22 (72.7%) FMCA	2000 17/24 (70.8%) FMCA	2009 50/65 (77.0%) FMCA	**0.008** ^a^	
Italy	Pisa/ Perugia/ Milan	Endocrine units of Pisa, Milan and Perugia [11]	1996-2000 21/56 (37.5%) DS	2001-2005 61/98 (62.2%) DS	2006-2010 56/106 (52.9%) DS			**0.012**
Korea	Seoul	Seoul St. Mary's Hospital [18, 19]	2008-2009 283/355 (79.7%) DS	2011-2012 165/200 (82.5%) DS			NS	
Ireland	Dublin	St. James's Hospital [20]	1982-1992 0/3 (0%) DS	1993-2002 11/15 (73.3%) DS			**0.043**	
Poland	Kielce	Holycross Cancer Center [21]	2000-2004 88/152 (57.9%) Three methods ^b^	2005 – 2009 140/195 (71.8%) Three methods ^b^	2010 – 2013 189/254 (74.4%) Three methods ^b^		**0.001** ^a^	

Abbreviations: NS, not significant; DS, direct sequencing; FMCA, fluorescent melting curve analysis

^a^ P value for trend adjusted for age and sex

^b^ All analyses that were performed prior to 2013 by DS and allele-specific PCR (AS-PCR) were verified by quantitative Real time PCR (qPCR). In total, the authors used qPCR to analyze BRAF mutations in 705/723 PTCs (97.5%). The majority of samples were tested using the three methods (DS, AS-PCR and qPCR).

We gathered enough information to determine the trends of *RET/PTC* rearrangements in classical PTCs at UCSF [13] and University of Pittsburgh [14], the University of Yamanashi (Japan) [19, 20] and St. James's Hospital (Ireland) [17]. There was a decreasing trend of *RET/PTC* rearrangements over time in classical PTCs at all these institutions (Table 3).

**Table 3:** The trends of *RET/PTC* rearrangements prevalence of classical PTCs from individual institutions

Country	City (State)	Institution (Reference)	Prevalence of *RET/PTC* rearrangements in classical PTCs				
			Period 1	Period 2	Period 3	Period 4	*P*
US	San Francisco (CA)	UCSF [13]	1991-1995 10/74 (13.5%) Nested PCR	1996-2000 19/103 (18.4%) Nested PCR	2001-2005 2/51 (3.9%) Nested PCR		**0.047**
US	Pittsburgh (PA)	Univ. of Pittsburgh [14]	1974-1985 6/52 (11.5%) DCI FISH	1990-1992 1/18 (5.6%) DCI FISH	2000 2/23 (8.7%) DCI FISH	2009 1/65 (1.5%) DCI FISH	NS
Japan	Yamanashi	Univ. of Yamanashi [22, 23]	2001-2006 4/14 (28.6%) Nested PCR	2011-2014 1/59 (1.7%) Nested PCR			**<0.001**
Ireland	Dublin	St. James's Hospital [20]	1982-1992 2/3 (66.7%) RT-PCR	1993-2002 4/15 (26.7%) RT-PCR			NS

Abbreviations: DCI FISH, dual colour interphase fluorescent *in situ* hybridization; RT-PCR, reverse transcription PCR

We could determine the trends for *RAS* mutations in classical PTCs at UCSF [13] and University of Pittsburgh [14]. The prevalence of *RAS* mutations was low and gradually decreased from 4.1% during the 1991-1995 period to 0% during the 2001-2005 period (*p* = 0.369). At the University of Pittsburgh, the RAS mutation prevalence was extremely low and remained stable over four periods (Table 4). *RAS* mutations are rarely detected in classical PTCs and therefore it can be difficult to observe the trend. As a result of this, we decided to further analyze the change of *RAS* mutations in FVPTCs from individual institutions. We could only gather information to determine the trend of *RAS* mutations in FVPTCs from University of Pittsburgh [14]. The prevalence of *RAS* mutations in FVPTCs was relatively stable during the first three periods but significantly increased from 11.1% in 2000 to 44.3% in 2009.

**Table 4:** Trends of *RAS* mutations prevalence of classical PTCs from individual institutions

Country	City (State)	Institution (Reference)	Prevalence of *RAS* rearrangements in classical PTCs				
			Period 1	Period 2	Period 3	Period 4	*P*
US	San Francisco (CA)	UCSF [13]	1991-1995 3/74 (4.1 %) DS	1996-2000 3/103 (2.9%) DS	2001-2005 0/51 (0%) DS		NS
US	Pittsburgh (PA)	Univ. of Pittsburgh [14]	1974-1985 0/52 (0.0%) FMCA	1990-1992 0/21 (0.0%) FMCA	2000 0.24 (0.0%) FMCA	2009 1/65 (1.5%) FMCA	NS

Abbreviations: DS, direct sequencing; FMCA, fluorescent melting curve analysis

All individual institutions applied the same protocol for mutational tests (same tissue treatment method, same DNA extraction method and same mutational detection method from same manufacturer) between different periods.

### Trends for *BRAF* mutations in micro PTCs from individual institutions

According to the WHO classification, micro PTCs are PTCs with a diameter ≤1cm. Data on the stratified list was eligible for selection in our review of micro PTCs if they met the same criteria as the criteria described above in classical PTCs section.

We could determine trends of the *BRAF* mutations in micro PTCs from four institutions. The *BRAF* mutation prevalence significantly increased at the Tianjin Medical University Cancer Hospital from 40.1% during 2001-2010 to 62.7% during 2013-2014, and investigators used direct sequencing for mutational analyses in both periods [21, 22]. Although the detection method for *BRAF* mutations was not disclosed within this study by Lu et al [22], the authors provided this information by email and confirmed that the PTC patients were recruited from Tianjin Medical University Cancer Hospital (an affiliated hospital of Tianjin Medical University). We also found the prevalence of *BRAF* mutations increased significantly at Samsung Medical Center [23, 24] and Holycross Cancer Center [18] (Table 5). At the University of Pittsburgh, prevalence of *BRAF* mutations in micro PTCs decreased from 32.3% during 1974-1985 to 18.8% during 1990-1992 and then gradually increased to 23.5% in 2000 and 38% in 2009 [14]. This change, however, was not statistically significant (*p* = 0.442).

**Table 5:** The trends of *BRAF* mutations prevalence of micro PTCs from individual institutions

Country	City (State)	Institution (Reference)	Prevalence of *BRAF* mutations in micro PTCs				
			Period 1	Period 2	Period 3	Period 4	*P*
US	Pittsburgh (PA)	Univ. of Pittsburgh [14]	1974-1985 10/31(32.3%) FMCA	1990-1992 3/16(18.8%) FMCA	2000 4/17 (23.5%) FMCA	2009 19/50(38%) FMCA	NS
Poland	Kielce	Holycross Cancer Center [21]	2000-2004 45/90 (50%) Three methods ^a^	2005-2009 104/147 (70.7) Three methods ^a^	2010-2013 147/203 (72.4) Three methods^a^		**0.001 ^b^**
Korea	Seoul	Samsung Medical Centre [26, 27]	2008-2009 253/396(63.9%) DS	2010-2011 97/128(75.8%) DS			**0.013**
China	Tianjin	Tianjin Medical Univ. Cancer Hospital [24, 25]	2001-2010 392/977(40.1%) DS	2013-2014 1244/1984(62.7%) DS			**<0.001**

Abbreviations: NS, not significant; DS, direct sequencing; FMCA, fluorescent melting curve analysis

^a^ All analyses that were performed prior to 2013 by DS and allele-specific PCR (AS-PCR) were verified by quantitative Real time PCR (qPCR). In total, the authors used qPCR to analyze *BRAF* mutations in 705/723 PTCs (97.5%). The majority of samples were tested using the three methods (DS, AS-PCR and qPCR).

^b^ P value for trend adjusted for age and sex

All included institutions used the same protocol for mutational detection among different surgical periods but one exception (Samsung Medical Center) in which the DNA materials were extracted from fine-needle aspirates in the former period and from paraffin-embedded tissues in the latter. However, all PCR products in these two periods were visualized on 2% agarose gel to confirm the good DNA quality prior to DNA purification step [23, 24], thus minimized the influence of technical issues on the results of direct sequencing.

### Trends of demographic and clinicopathological features and molecular profiles in PTCs over time

To avoid bias, we only included articles with consecutive or random selection of patients and no criteria used in patient selection. We evaluated these trends only within individual institutions. We also excluded articles with consecutive or random selection of patients, but which focused on particular characteristics such as classical PTCs or patients who underwent total thyroidectomy or radioiodine ablation (possible bias toward aggressive PTCs and therefore could influence the nature of the PTC cohort). Five single- or multi-institutions met our criteria: University of Pittsburgh [14], University of Padua [25, 26], multicenter study from Pisa/Perugia/Milan [11], Samsung Medical Centre [24, 27] and Holycross Cancer Center [18].

Patients’ ages at time of diagnosis increased during the most recent period at all included institutions. Another important finding is the significant increase in the proportion of micro carcinomas at all included institutions. The rates of ETE and lymph node metastasis (LNM) decreased at most of the institutions. The rate of multifocal PTCs stayed relatively stable over time (data not shown). Table 6 describes changes in demographic data and clinicopathological features of PTCs over time.

**Table 6:** Modifications of demographic and clinicopathological characteristics of PTCs over time from individual institutions

Country	Institution (Reference)	Period	Patients’ age (years)	*p*	Male (%)	*p*	Tumor size (%)	*p*	FVPTC (%)	*p*	ETE (%)	*p*	LNM (%)	*p*
US	University of Pittsburgh[14]	1974-1985 1990-1992 2000 2009	Median, 37 Median, 37 Median, 50 Median, 53	**<0.001**	27.6 27.1 13.2 24.5	NS	≤1cm, 42.0 ≤1cm, 44.1 ≤1cm, 41.5 ≤1cm, 51.3	NS	10.2 3.4 13.2 25.2	**<0.001**	40.2 27.1 26.9 20.9	**0.005**	26.8 28.8 20.8 18.3	**<0.001**
Italy	University of Padua[28, 29]	2005-2007 2008-2009	Mean, 43.6* Mean, 49.0	**0.003**	28.9 22.7	NS	ND ≤1cm, 24.9	NA	3.6 3.5	NS	ND 45.4	NA	51.8 38.3	**0.049**
Italy	Endocrine units of Pisa/Perugia/ Milan [11]	1996-2000 2001-2005 2006-2010	Mean, 40.9 Mean, 44.0 Mean, 47.6	**0.014**	32.0 25.0 32.7	NS	<2cm, 43.3 <2cm, 59.4 <2cm, 61.0	**0.01**	12.3 18.4 15.3	NS	T3/T4, 45 T3/T4, 39 T3/T4, 35	**0.005**	50.7 48.1 51.9	NS
Poland	Holycross Cancer Center [21]	2000-2004 2005-2009 2010-2013	Median, 50 Median, 53 Median, 53	NS	11.3 11.6 13.1	NS	≤1cm, 50.8 ≤1cm, 63.1 ≤1cm, 64.9	**0.003**	11.3 12.9 15.0	NS	22.5 25.3 24.9	NS	12.4 12.9 13.7	NS
Korea	Samsung Medical Centre[27, 30]	2008 2010-2011	Mean, 47.0 Mean, 48.0	NS	18.5 16.9	NS	≤1cm, 55.6 ≤1cm, 80.5	**<0.001**	2.9 ND	NA	62.2 53.5	NS	48.1 36.5	**0.043**

Abbreviations: FVPTC, follicular variant papillary thyroid carcinoma; ETE, extrathyroidal extension; LNM, Lymph node metastasis; NS, not significant; NA, not available; ND, not described

* Estimated the sample mean and standard deviation as described by Hozo *et al*. [16]

### Quality assessment and risk of bias analysis

The quality score ranged from 40% to 70%, as measured by the NIH Quality Assessment Tool for Observational Cohort and Cross-Sectional Studies [28]. The lowest scored was 40% (Nakazawa et al. [19]) and the highest score was 70% (Jung et al. [14]). Quality of all included articles were classified as fair, except the two studies by Smyth et al. [17] and Nakazawa et al. [19] which were regarded as low quality. Most of included studies lacked sample size justification. Quality scores for each NIH criteria are shown in Supplementary Table.

## DISCUSSION

The incidence of thyroid cancer has been increasing at an amazing rate in recent decades, especially PTCs [3, 4]. There is still debate as to whether improved detection is the only reason for the increase of PTCs or whether the tumorigenesis of thyroid cancer has changed [3, 6]. From our findings, the basic nature of PTC has been changing in recent decades (Table 6). The increase in the proportion of microcarcinomas that are diagnosed is associated with the improved detection rate in recent years, especially the development of ultrasonography. The majority of microcarcinomas show indolent behaviors, which can explain our findings that PTCs manifested less aggressive behaviors in the most recent periods we analyzed.

With improved screening and detection methods, it is reasonable to believe that patients would be diagnosed at an earlier age. However, our analysis suggests the opposite result. A number of publications showed that *BRAF* mutations are found in a higher proportion of older patients [12, 29–34], and *RET/PTC* rearrangements are commonly found in young patients [35–37]. Our systematic review demonstrated an increased prevalence of *BRAF* mutations (typically older patients) accompanied by a decreased prevalence of *RET/PTC* rearrangements (typically young patients).

Other than the multicenter study from the endocrine units of Pisa/Perugia/Milan [11] and the study from Holycross Cancer Center [18], the male proportion of patients with thyroid cancer has been decreasing over the years, although the difference was not significant (Table 6). Recent data showed that the increased incidence in thyroid cancer has affected more women than men, which supports our findings [3, 4].

There is still the question as to why the prevalence of *BRAF* mutations has been increasing significantly and the prevalence of *RET/PTC* rearrangements has been falling over the years. It is difficult to address the possible reasons for these changes because there are no known etiologies or risk factors that could trigger or activate these genetic alterations in PTCs other than radiation-induced *RET/PTC* [38]. From our findings, the significant increase of *BRAF* mutations in classical PTCs has occurred in different geographical areas (Table 2). Interestingly, we also found a remarkable increase of *BRAF* mutations in micro PTCs from Kielce, Seoul and Tianjin (Table 5). Elisei et al. suggested worldwide iodine supplementation as a potential candidate for the worldwide increasing trend of *BRAF* mutations [39]. How iodine intake may influence *BRAF* mutations in PTCs, however, is still controversial. Guan et al. reported that high iodine intake was associated with a higher prevalence of the *BRAF V600E* mutation in Chinese PTCs [40]. To the best of our knowledge, this is the only publication that linked iodine intake with occurrence of *BRAF* mutations. Frasca et al. reported there was no statistical difference in *BRAF V600E* prevalence in Italian PTCs from iodine-sufficient *vs*. iodine-deficient areas [8]. Data collected from the US showed that the US population has sufficient iodine intake [41, 42]. Because of this, it is irrational to correlate the increasing trend of *BRAF* mutations in classical PTCs seen in California and Pittsburgh with iodine intake. In our recent study, we found no statistical significance in the prevalence of *BRAF V600E, RET/PTC* rearrangements or *RAS* mutations in papillary and follicular thyroid carcinomas from an iodine-rich country (Japan) and an iodine-deficient country (Vietnam) [20]. Research in recent decades has increased our understanding of the molecular pathogenesis of thyroid cancer [1, 10, 43]. However, the origin of *BRAF* mutations in thyroid cancer still needs clarification. Further experimental studies should better clarify the origin of these mutations and help determine the reasons behind the increase in *BRAF* mutations over the last few decades.

A significant decrease of *RET/PTC* rearrangements in classical PTCs has been seen over the years (Tables 3). *RET/PTC* rearrangements have been strongly associated with radiation-induced PTCs [35, 44, 45]. *RET/PTC* rearrangements could also be detected in sporadic PTCs, but the underlying causes are unknown. A worldwide decrease in exposure to ionizing radiation in recent years, long after the Chernobyl accident or nuclear test experiments, has been proposed as a possible reason for the decrease of *RET/PTC* rearrangements [39]. Another hypothesis is that *RET/PTC* rearrangements are induced by unknown determinants that have been changing over the last decades [39]. We are in favor of the latter hypothesis because there were no nuclear accidents or atomic bomb experiments in the UK, Italy or Japan that could explain the high prevalence of *RET/PTC* rearrangements in sporadic PTCs in the earlier period of our review. In recent years, there has been another nuclear accident in Fukushima (Japan) in March 2011. *BRAF* mutations were the major genetic event (63.2%) in pediatric PTCs in Fukushima who underwent thyroidectomy in 2013 - 2014 and *RET/PTC* rearrangements were found in only 10.3% of cases [46]. This oncogenic profile is quite different from Chernobyl-related studies [47] but this result should be interpreted with cautions. Firstly, the thyroid equivalent doses in Fukushima (4.2 mSV) is much lower than in Chernobyl (490 mSV) [48, 49]. The second issue is that at the time of the study by Mitsutake et al. [46], the interval period from the date of accident to the onset was too short. Continuous studies are required to further investigate the trends of thyroid cancer incidence and genetic alterations in Japanese children.

At the University of Pittsburgh, the frequency of *RAS* mutations in PTCs remained low and stable during 1974-2000, then significantly increased in 2009 [14]. This trend was accompanied by a remarkable increase of FVPTCs in 2009 (Table 6). The incidence of FVPTC was reported to have increased nearly three-fold from 1973 to 2003 in the US [50]. Most of the increase in FVPTCs occurred after 1988 when the WHO classification changed. However, the proportion of FVPTCs did not change in Italy over the years [11]. We can only examine the trend of *RAS* mutations in classical PTCs from UCSF and University of Pittsburgh. The prevalence of *RAS* mutations did not change in classical PTCs from the University of Pittsburgh over the four periods in our review, but its prevalence in FVPTCs did increase significantly from 11.1% in 2000 to 44.3% in 2009 [14]. The authors from UCSF only selected patients with classical PTCs for their study, and they identified *RAS* mutations in 4%, 2.9% and 0% of cases during 1991 to 1995, 1996 to 2000 and 2001 to 2005, respectively (Table 4). Further studies should examine the trend of *RAS* mutations in PTCs, especially the follicular variant or follicular neoplasms (follicular thyroid adenoma and carcinoma).

Our systematic review is limited by the relatively small number of institutions that can be included. However, we must apply strict criteria as described above to avoid the potential bias caused by heterogeneities in population characteristics, detection methods and other factors that could influence the prevalence of genetic alterations. Further population-based studies with large number of cases are required to clarify these trends. In addition, we could not perform multivariate analysis for trends of mutations adjusted for the confounding factors because most of included studies did not provide those data or individual patient data. Jung et al. and Kowalska et al. investigated the trend of mutations in classical PTCs adjusted for age and sex and they found a significant increase of *BRAF* mutations over time [14, 18] (Table 2).

From our study, it is evident that the demographic and clinicopathological characteristics and the molecular profiles of PTCs have been changing over the years worldwide, suggesting the changes in tumorigenesis of PTCs. Further research on whether the genetic alterations in PTCs are primary or secondary and elucidation of molecular pathways in the pathogenesis of thyroid cancer can help us clarify the underlying causes of these modifications.

## MATERIALS AND METHODS

### Search strategy and study identification

We conducted an electronic search within six databases (PubMed, Scopus, ISI Web of Science, Google Scholar, WHO Global Health Library and Virtual Health Library) to identify relevant articles using the search term: (papillary thyroid AND (carcinoma OR cancer) AND (BRAF OR RAS OR RET/PTC). We did not limit the search results as to language or year of publication. Our study included publications published before June 2015. Additionally, we performed a manual search by reviewing citations within the included publications and related references presented in PubMed. Our study strictly followed the recommendation of Preferred Reporting Items for Systematic Review and Meta-analysis (PRISMA) statement [51]. We registered our study protocol at PROSPERO database: http://www.crd.york.ac.uk/prospero/display_record.asp?ID=RD42015024778.

### Selection criteria

We imported search results from 6 libraries into Endnote (Thompson Reuters) and deleted duplicates. Two teams of reviewers independently screened the abstracts using the predetermined selection criteria. The inclusion criteria were (i) articles containing data regarding frequencies of *BRAF* mutations, *RET/PTC* rearrangements or *RAS* mutations in PTCs or classical PTCs which had been histologically confirmed, (ii) articles containing data regarding frequencies of *RAS* mutations in follicular variant PTCs (FVPTCs) or (iii) articles comparing the clinicopathological characteristics of PTCs between different time periods. We excluded studies that (i) did not provide the locations or institutions where the patients with PTC were recruited or treated, (ii) focused only on a histological variant (with two exceptions described in the inclusion criteria) or a clinicopathological factor (e.g. pediatric PTCs, multifocal PTCs or recurrent PTCs), (iii) involved radiation-induced PTCs unless the study had a mixed population of radiation-induced and sporadic PTCs with the sporadic PTC data reported separately, (iv) were experimental or *in vitro* in nature, (v) were case reports, conference presentations, posters, proceeding papers, books, theses or reviews or (vi) duplicated data. If a study's authors did not indicate where patients’ data was collected or where they were treated and all authors were from the same affiliation, the affiliation location was considered the primary place where the patients were initially treated.

### Data extraction

Two teams of reviewers independently read the full text of potential articles and extracted data using a predefined data extraction form. Supervisors resolved any discrepancies during this data extraction step. We extracted the following details: name of institution, city, country, time of surgery (when the patient was admitted), patient's age and gender, patient selection method, mutational detection method, prevalence of genetic alterations in entire PTC population and each histological variant, and clinicopathological data (histological variants, size, multiplicity, extrathyroidal extension (ETE) and TNM classification). We sought missing data from potentially eligible studies *via* email with the authors. If the full text did not indicate when patients received surgery and the authors did not respond to our emails, we considered time before submission date of the article as a patient's surgical time period. Native speakers from a group of international doctors extracted data from articles not written in English, and this was further checked by Google Translate.

### Data analysis

Our systematic literature review assessed several factors: 1) *BRAF, RET/PTC* and *RAS* mutational trends in classical PTCs from individual institutions; 2) *BRAF* mutational trends in micro PTCs from individual institutions; 3) modifications and trends of clinicopathological characteristics of PTCs from individual institutions. Selection and analysis of data for each section is described in detail below.

We compared the means and standard deviations (SD) of age and tumor size by using Student *t* test in GraphPad QuickCals at http://www.graphpad.com/quickcalcs/ttest1.cfm?Format=SD For those articles in which the authors only provided median, range and the total number of patients, we estimated mean and SD values as described by Hozo et al [52]. Pearson Chi-square was used to compare the clinicopathological features and prevalence of each mutation between different periods from the same institutions. Statistical analysis was performed with the SPSS software, version 22 (Chicago, IL). A *p* value ≤ 0.05 was considered statistically significant.

### Quality assessment and risk of bias analysis

Two investigators independently scored the quality and risk of bias of the included studies, as measured by the Quality Assessment Tool for Observational Cohort and Cross-Sectional Studies of the National Institute of Health (NIH) [28]. Disagreements in scoring was solved by discussion and consensus. The score of all 14 items marked with “yes” were calculated into percentage (inapplicable items were not taken into account). The quality rating of included studies was classified as low, fair and good if the score percentage was lower than 50%, 50% - 75% and more than 75%, respectively.

## SUPPLEMENTARY TABLE


