# Supplementary material for: The changing characteristics and molecular profiles of papillary thyroid carcinoma over time: a systematic review
Source: Oncotarget. 2016 Oct 25;8(6):10637–49. doi: 10.18632/oncotarget.12885 (PMC5354688; doi:10.18632/oncotarget.12885)
Supplement: Supplementary file 1 [file oncotarget-08-10637-s001.pdf]

The changing characteristics and molecular profiles of papillary thyroid carcinoma over time - a systematic review

Supplementary Material

Supplementary Table. Risk of bias assessment of included studies

Criteria	Mathur et al. [1]	Jung et al. [2]	Romei et al. [3]	Nam et al. [4]	Lee et al. [5]	Smyth et al. [6]	Nakazawa et al. [7]	Vuong et al. [8]	Lee et al. [9]	Kang et al. [10]	Zheng et al. [11]	Lu et al. [12]	Barzon et al. [13]	Pelizzo et al. [14]	Hwang et al. [15]	Kowalska et al. [16]
1. Was the research question or objectives in this paper clearly stated	Yes	Yes	Yes	Yes	Yes	Yes	Yes	Yes	Yes	Yes	Yes	Yes	Yes	Yes	Yes	Yes
2. Was the study population clearly specified and defined?	Yes	Yes	Yes	Yes	Yes	Yes	No	Yes	Yes	Yes	Yes	Yes	Yes	Yes	Yes	Yes
3. Was the participation rate of eligible persons at least 50%?	Yes	Yes	Yes	Yes	Yes	CD	CD	Yes	Yes	Yes	Yes	Yes	Yes	Yes	Yes	Yes
4. Were all the subjects selected or recruited from the same or similar populations? Were inclusion and exclusion criteria for being in the study pre-specified and applied uniformly to all participants?	Yes	Yes	Yes	Yes	Yes	Yes	Yes	Yes	Yes	Yes	Yes	Yes	Yes	Yes	Yes	Yes
5. Was a sample size justification, power description, or variance and effect estimates provided?	No	Yes	No	No	No	No	No	No	No	No	No	No	No	No	No	No
6. For the analyses in this paper, were the exposures of interest measured prior to the outcomes being measured?	NR	Yes	Yes	NR	NR	NR	Yes	NR	NR	NR	NR	NR	NR	NR	NR	Yes
7. Was the timeframe sufficient so that one could reasonably expect to see an association between exposure and outcome if it existed?	NA	NA	NA	NA	NA	NA	NA	NA	NA	NA	NA	NA	NA	NA	NA	NA
8. For exposures that can vary in amount or level, did the study examine different levels of the exposure as related to the outcome (e.g., categories of exposure, or exposure measured as continuous variable)?	NA	NA	NA	NA	NA	NA	NA	NA	NA	NA	NA	NA	NA	NA	NA	NA
9. Were the exposure measures (independent variables) clearly defined, valid, reliable, and implemented consistently across all study participants?	No	No	No	No	No	No	No	No	No	Yes	No	No	No	No	No	No
10. Were the exposures assessed	NA	NR	NR	NA	NA	NA	NR	NA	NA	NA	NA	NA	NA	NA	NA	NR

more than once over time?																
11. Were the outcome measures (dependent variables) clearly defined, valid, reliable, and implemented consistently across all study participants?	Yes	Yes	Yes	Yes	Yes	Yes	Yes	Yes	Yes	Yes	Yes	Yes	Yes	Yes	Yes	Yes
12. Were the outcome assessors blinded to the exposure status of participants?	NR	NR	NR	NR	NR	NR	NR	NR	NR	NR	NR	NR	NR	NR	NR	NR
13. Was loss to follow-up after baseline 20% or less?	NA	NA	NA	NA	NA	NA	NA	NA	NA	NA	NA	NA	NA	NA	NA	NA
14. Were key potential confounding variables measured and adjusted statistically for their impact on the relationship between exposures and outcomes?	NA	NA	NA	NA	NA	NA	NA	NA	NA	NA	NA	NA	NA	NA	NA	NA
**NIH (%)[a]**	**56**	**70**	**60**	**56**	**56**	**44**	**40**	**56**	**56**	**56**	**56**	**56**	**56**	**56**	**56**	**60**
**Quality rating**	**Fair**	**Fair**	**Fair**	**Fair**	**Fair**	**Low**	**Low**	**Fair**	**Fair**	**Fair**	**Fair**	**Fair**	**Fair**	**Fair**	**Fair**	**Fair**

Abbreviations: NA, not applicable; NR, not reported, CD, cannot determine

[a] The score of all 14 items marked with “yes” were calculated into percentage (inapplicable items were not taken into account)

Supplementary References

1. Marthur A, Moses W, Rahbari R, Khanafshar E, Duh QY, Clark O, Kebebew E. Higher rate of BRAF mutation in papillary thyroid cancer over time: a single-institution study. Cancer. 2011; 117: 4390-5. doi:

2. Jung CK, Little MP, Lubin JH, Brenner AV, Wells SA, Jr., Sigurdson AJ, Nikiforov YE. The Increase in Thyroid Cancer Incidence During the Last Four Decades Is Accompanied by a High Frequency of BRAF Mutations and a Sharp Increase in RAS Mutations. J Clin Endocrinol Metab. 2014; 99: E276-E85. doi:

3. Romei C, Fugazzola L, Puxxedu E, Frasca F, Viola D, Muzza M, Moretti S, Nicolosi ML, Giani C, Cirello V, Avenia N, Rossi S, Vitti P, et al. Modifications in Papillary Thyroid Cancer gene profile Over the Last 15 Years. J Clin Endocrinol Metab. 2012; 97: 1758-65. doi:

4. Nam JK, Jung CK, Song BJ, Lim DJ, Chae BJ, Lee NS, Park WC, Kim JS, Jung SS, Bae JS. Is the BRAF(V600E) mutation useful as a predictor of preoperative risk in papillary thyroid cancer? Am J Surg. 2012; 203: 436-41. doi:

5. Lee SH, Jung CK, Bae JS, Jung SL, Choi YJ, Kang CS. Liquid-based cytology improves preoperative diagnostic accuracy of the tall cell variant of papillary thyroid carcinoma. Diagn Cytopathol. 2014; 42: 11-7. doi:

6. Smyth P, Finn S, Cahill S, O'Regan E, Flavin R, O'Leary JJ, Sheils O. ret/PTC and BRAF act as distinct molecular, time-dependant triggers in a sporadic Irish cohort of papillary thyroid carcinoma. Int J Surg Pathol. 2005; 13: 1-8. doi:

7. Nakazawa T, Murata S-i, Kondo T, Niu D, Mochizuki K, Kawasaki T, Yamane T, Nakamura N, Katoh R. RET/PTC rearrangements arising from a small population of papillary thyroid carcinoma cells, possible candidate for passenger mutation. Virchows Archiv. 2009; 455: 35-41. doi:

8. Vuong HG, Kondo T, Oishi N, Nakazawa T, Mochizuki K, Inoue T, Kasai K, Tahara I, Tran TM, Hirokawa M, Katoh R. Genetic alterations of differentiated thyroid carcinoma in iodine-rich and iodine-deficient countries. Cancer Med. 2016. doi:

9. Lee JI, Jang HW, Kim SW, Kim J-W, Oh YL, Chung JH. BRAF(V600E) mutation in fine-needle aspiration aspirates: Association with poorer prognostic factors in larger papillary thyroid carcinomas. Head Neck. 2013; 35: 548-53. doi:

10. Kang SY, Ahn S, Lee S-M, Jeong JY, Sung J-Y, Oh YL, Kim K-M. Shifted termination assay (STA) fragment analysis to detect BRAF V600 mutations in papillary thyroid carcinomas. Diagn Pathol. 2013; 8. doi:

11. Zheng X, Wei S, Han Y, Li Y, Yu Y, Yun X, Ren X, Gao M. Papillary microcarcinoma of the thyroid: clinical characteristics and BRAF(V600E) mutational status of 977 cases. Ann Surg Oncol. 2013; 20: 2266-73. doi: 10.1245/s10434-012-2851-z.

12. Lu ZZ, Zhang Y, Wei SF, Li DS, Zhu QH, Sun SJ, Li M, Li LI. Outcome of papillary thyroid microcarcinoma: Study of 1,990 cases. Mol Clin Oncol. 2015; 3: 672-6. doi:

13. Barzon L, Masi G, Boschin IM, Lavezzo E, Pacenti M, Ide EC, Toniato A, Toppo S, Palu G, Pelizzo MR. Characterization of a novel complex BRAF mutation in a follicular variant papillary thyroid carcinoma. Eur J Endocrinol. 2008; 159: 77-80. doi:

14. Pelizzo MR, Boschin IM, Barollo S, Pennelli G, Toniato A, Zambonin L, Vianello F, Piotto A, Ide EC, Pagetta C, Sorgato N, Torresan F, Girelli ME, et al. BRAF analysis by fine needle aspiration biopsy of thyroid nodules improves preoperative identification

of papillary thyroid carcinoma and represents a prognostic factor. A mono-institutional experience. Clin Chem Lab Med. 2011; 49: 325-9. doi:

15. Hwang J, Shin JH, Han B-K, Ko EY, Kang SS, Kim J-W, Chung JH. Papillary Thyroid Carcinoma With BRAF(V600E) Mutation: Sonographic Prediction. AJR Am J Roentgenol. 2010; 194: W425-W30. doi:

16. Kowalska A, Walczyk A, Kowalik A, Palyga I, Trybek T, Kopczynski J, Kajor M, Chrapek M, Pieciak L, Chlopek M, Gozdz S, Kaminski G. Increase in Papillary Thyroid Cancer Incidence Is Accompanied by Changes in the Frequency of the BRAF(V600E) Mutation: A Single-Institution Study. 2016. doi:
